# Zinc Ferrite Nanoparticle Coatings on Austenitic Alloy Steel

**DOI:** 10.3390/ma17040857

**Published:** 2024-02-12

**Authors:** Martin Ochmann, Libor Machala, Miroslav Mašláň, Vítězslav Heger, Tomáš Krátký

**Affiliations:** 1 Department of Experimental Physics, Faculty of Science, Palacký University, 17. listopadu 1192/12, 77900 Olomouc, Czech Republic; martin.ochmann01@upol.cz (M.O.); miroslav.maslan@upol.cz (M.M.); vitezslav.heger@upol.cz (V.H.); 2 Centre of Hydraulic Research, 78349 Lutín, Czech Republic; t.kratky@sigma.cz

**Keywords:** austenitic alloy steel, zinc ferrite, covering layer, nanoparticles, annealing

## Abstract

The phase transition of austenitic stainless steel of commercial label CL20ES and zinc ferrite nanoparticles was studied in an oxidative atmosphere of dry air to develop a low-cost, effective technique for covering-layer fabrication. CL20ES powder and zinc ferrite powder were mechanically mixed. This mixture was studied in an atmosphere of dry air at different annealing temperatures from room temperature to 900 °C. The employed characterization techniques are X-ray powder diffraction, Mössbauer spectroscopy in the transmission geometry, and scanning electron microscopy with elemental mapping. The fabricated layers were also characterized by surface-specific techniques such as conversion electron Mössbauer spectroscopy and grazing incidence X-ray powder diffraction. The analyzed powder mixture shows resistance against oxidation in dry air and high temperatures. These results were employed to produce zinc ferrite covering layers on 3D-printed cylinders of CL20ES. The results show a predisposition of zinc ferrite to be recrystallized at temperatures above 350 °C without the production of corrosive substances on steel. The zinc ferrite layers were analyzed by an ultrasonic hardness tester as well, which proved the hardness enhancement.

## 1. Introduction

The ongoing progress in almost all human activities increases the requirements and demand for commonly used materials, steel included. Thus, new types of materials, enhancement, or fabrication processes are constantly being developed to provide materials with parameters to match the requirements of specific applications, e.g., high corrosion resistivity, excellent mechanical properties, etc. [1,2,3,4,5]. Alloy steels are a type of steel that contain additional alloying elements other than carbon, e.g., chromium, nickel, molybdenum, or manganese. The alloy steel composition is designed to increase the steel’s resistivity against environmental degradation (i.e., acids, hydroxides, or oxidizing agents) in comparison to classic steels, while preserving or even enhancing their tensile strength and hardness [6]. Nonetheless, the alloying elements, which can be present in relatively high amounts (especially chromium and nickel), also increase the economic cost of the said steels [7,8]. Moreover, these types of steel can have iron in the structural form of austenite (γ-Fe), thus forming so-called austenitic steel. However, austenite is not generally a thermodynamically stable phase of steel and is stabilized by alloying elements. Stainless steel contains at least 7% chromium, while austenite stabilization requires at least 16% chromium, according to [9]. Chromium in the surface layers forms a passive film in the presence of oxygen. This film protects iron from iron oxide formation like hematite. It has been shown that high-temperature phase transformations can lead to the loss of stainless steel’s anti-corrosive properties [10]. For example, the commercial CL20ES austenitic steel loses its stainless properties at around 700 °C [11], where intercrystallite rusting appears. The presence of chromium in the bulk does not completely prevent rust formation in the surface layer, as investigated by [12,13,14,15]. These facts, combined with a higher price of alloy steel, have stimulated the utilization of various enhancing surface treatments, which could additionally eliminate chromium. The high amount of chromium limits the application of surface treating methods. Classical approaches, which remain in researchers’ interest, are based on the preparation of conversion layers, such as phosphating, bluing, or controlled oxidation [16,17,18,19,20,21]. However, these processes (especially the hot bath methods) often involve environmentally hazardous materials, either in the layer itself or during the production phase. Thus, in order to eliminate the environmental impact, there is a need to modify these processes or replace the harmful substances, e.g., chromium, as described in [22,23,24]. New strategies for a low-cost, green, and tunable steel surface enhancement need to be developed. One of the promising solutions could be the deposition of an oxide or double hydroxide protective layer of defined chemical composition [25,26,27,28]. The motivation is to develop a procedure that allows us to form a covering layer even on a high-chromium steel, because other effective techniques are very limited.

Cubic spinel ferrites, e.g., zinc ferrite, are a family of chemical compounds with the formula MeFe_2_O_4_, where Me is a divalent metal cation (i.e., Fe, Mg, Zn, Ni, Cu, or Co). Among these elements, only iron and cobalt can be present in divalent and trivalent form. Most of the spinel ferrites are thermodynamically very stable [29]. As oxides with metal cations already possess high oxidation numbers, they usually cannot be oxidized further. Cubic spinels are a part of the Fd-3m space group. Fd-3m is a face-centered cubic structure which is similar to the crystallite structure of austenite. Austenite ($\begin{array}{c} \gamma -Fe \end{array}$) is face-centered cubic and alpha iron has a body-centered cubic structure. Cubic ferrites are commonly known for their magnetic properties, where most ferrites are ferrimagnetic; thus, they are suitable for magnetic applications such as cores of coils or magnetic separation. On the other hand, zinc ferrite is an antiferromagnetic ferrite, which could also be superparamagnetic when nanosized [30]. The non-magnetic macroscopic behavior of zinc ferrite is similar to that of austenite; both of them demonstrate a doublet spectral component in the Mössbauer spectra. Alpha iron is ferromagnetic with a sextet spectral component in Mössbauer spectroscopy and is used as a calibration material for this technique. The common synthesis procedures include, for example, a solid-state synthesis and coprecipitation via alkalization of divalent metal cations and ferric ions. A purely solvothermal synthesis usually provides ferrite nanoparticles with a low crystallinity. On the other hand, a better crystallinity, but a lower specific surface area, is reached with a solid-state calcination at high temperatures (typically from 900 °C to 1200 °C).

The first preliminary study was based on the synthesis of zinc ferrite under different conditions [31]. The synthetic procedure is used with identical synthetic parameters. Among different samples suitable for these experiments, only the most simple procedure was chosen to prove the viability of the layer fabrication. In another study, we showed that zinc ferrite deposited on CL20ES plates can provide increased hardness by 9% [32]. In this study, we further investigate the possibilities of enhancing the properties of CL20ES austenitic alloy steel powder with a zinc ferrite (ZnFe_2_O_4_) surface layer towards higher hardness and higher chemical stability. CL20ES is the austenitic alloy steel powder of choice for selective laser 3D printing technology, which provides printed parts with good mechanical properties [33]. However, this type of steel is prone to surface oxidation during the 3D printing process. The surface metal oxides were detected even when the 3D printing process was carried out in an inert atmosphere [10].

The current study is divided into two sections: (i) investigation of the potential solid-state reaction between the zinc ferrite and CL20ES austenitic steel, which could promote the formation of iron corrosive phases (iron oxides or oxohydroxides), and (ii) deposition and subsequent investigation of the zinc ferrite layer on the CL20ES 3D-printed plates (d=25 mm×25 mm×3 mm), replaced subsequently by cylinders (d=25 mm, h=5.0 mm) calcined at different temperatures. The samples were studied with in situ high-temperature X-ray powder diffraction (XRD), X-ray powder diffraction in a grazing incidence mode, ^57^Fe Mössbauer spectroscopy, both in transmission (TMS) and conversion electron mode (CEMS), and optical and scanning electron microscopy. We believe the current study brings further insight into the oxide protective coatings of steels. Additionally, as steel is not the only alloy prone to corrosive oxidation, similar technological solutions could be implemented in, for example, magnesium or aluminum alloys as well [34,35,36].

## 2. Materials and Methods

### 2.1. Zinc Ferrite Nanoparticles

**Figure 1:**
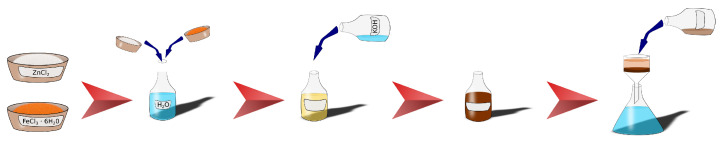
Simplified scheme of the synthetic procedure of zinc ferrite nanoparticle preparation.

The preparation of zinc ferrite nanoparticles was carried out according to the protocol that was published in our previous article [31]. The details of the preparation and characterization of the zinc ferrite nanoparticles can be found in the article [31]. The chemical compounds ZnCl_2_ (p.a.), FeCl_3_·6H_2_O (p.a.), and KOH (99%) were used for the synthesis and were purchased from PENTA, s.r.o. (Prague, Czech Republic). Briefly, the nanoparticles were synthetized by co-precipitation of Zn^2+^ and Fe^3+^ with a solution of KOH. After 60 min of vigorous stirring, the reaction was stopped and the supernatant liquid removed by vacuum filtration. The prepared ZnFe_2_O_4_ nanoparticles were washed several times with deionized water and left to dry in ambient atmosphere. The zinc ferrite sample, denoted as RT-60 (according to the synthetic parameters; 60 min, room temperature), was used in all following experiments. The preparation scheme is depicted in Figure 1.

**Figure 2:**
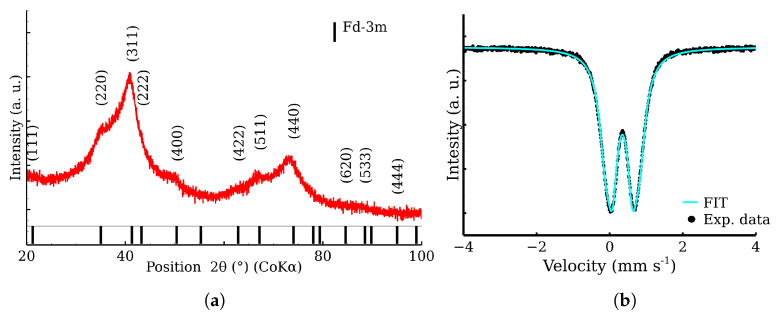
Checkout analysis of zinc ferrite sample. (**a**) XRD pattern of the sample; (**b**) Transmission Mössbauer spectrum of the sample.

The XRD analysis determined a mean coherent length of 2.9 nm by XRD and excluded other crystalline phases. Using TEM, the size of the prepared nanoparticles was found to be under 5 nm. The zinc ferrite nanoparticles exhibited a large BET area (220 m^2^ g^−1^) and pore volume. The sample contains highly aggregated nanoparticles with a mean size of aggregates of 85 nm, visually proved by scanning electron microscopy in transmission mode. The phase and elemental purity were checked by XRD, Mössbauer spectroscopy, and energy dispersive spectroscopy. The zinc ferrite is normal cubic spinel with ratio of 1 zinc atom to 2 iron atoms. The nanoparticles were found to be superparamagnetic, as determined by the room-temperature and the low-temperature Mössbauer spectroscopy and low-temperature magnetometric measurements. A representative sample was checked by XRD and Mössbauer spectroscopy; see Figure 2. The broadened diffraction peaks and the Fd-3m structure confirmed the presence of the nanoscaled zinc ferrite. The doublet is a characteristic spectral component of non-magnetic iron compounds, including superparamagnetic nanoparticles [31].

### 2.2. Austenitic Stainless Steel CL20ES

The austenitic stainless steel powder used in the experiments has the commercial name Cl20ES. The commercially available austenitic stainless steel powder Cl20ES (Cleveland-Cliffs Inc., Cleveland, OH, USA) was used in all the experiments as purchased. Particles have a size of approximately 50 μm, and they are mostly spherical. The chemical composition is listed in Table 1.

### 2.3. CL20ES and ZnFe_2_O_4_ Mixture

The prepared mechanical mixture of CL20ES and ZnFe_2_O_4_ contained 100 mg of ZnFe_2_O_4_ and 500 mg of CL20ES. A mechanical mixture of CL20ES and ZnFe_2_O_4_ was isothermally calcined in a muffle furnace LE 05/11 HT 40P (LAC, Rajhrad, Czech Republic) for 2 h in dry air at different temperatures. The initial temperature rate was 3000 °C h^−1^. The heating temperatures were 300 °C, 400 °C, 500 °C, 600 °C, 800 °C, and 900 °C. After heating, the samples were left to cool down at room temperature. The analyses of all listed samples were performed under laboratory conditions.

### 2.4. Fabrication of ZnFe_2_O_4_ Covering Layer

CL20ES cylinders and plates were fabricated by laser additive technology (SLM) under the protective atmosphere of the inert gas (argon). These samples were thermally treated at 550 °C in the air for 50 h after laser sintering to reduce the tension. Finally, the fabricated parts were sandblasted to eliminate the hematite in the surface layers. The procedure is described in detail in [10].

**Figure 3:**
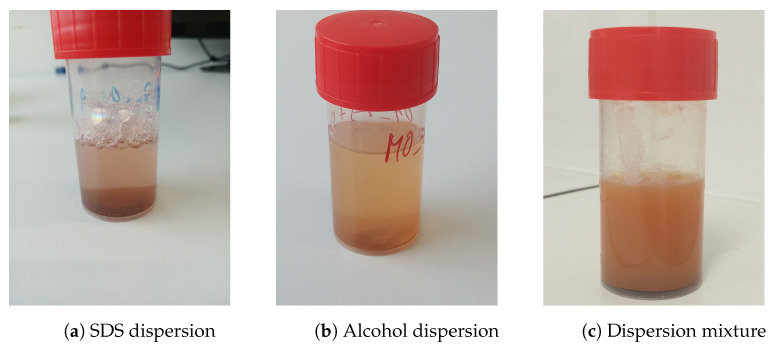
Zinc ferrite nanoparticles dispersed in various organic solvents and/or deionized water.

**Figure 4:**
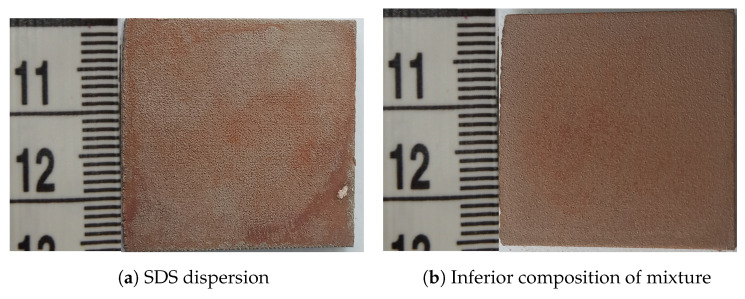
Samples of CL20ES plates with the zinc ferrite covering film dispersed in different solutions. The dimensions of the plate are 25 mm×25 mm×3 mm.

The dispersion of the zinc ferrite nanoparticles was prepared by the following procedure. The nanoparticles of zinc ferrite powder (80 mg) were dispersed in a mixture of deionized water (17.0 mL), isopropyl alcohol (4.0 mL), acetone (4.0 mL), and ethylene glycol (5.0 mL), assisted by an ultrasonic field. The ultrasonic field of low intensity was applied for an hour to ensure an optimal dispersion without any induced reactions. The composition and solvent volumes were chosen according to the relative level of dispersion of zinc ferrite and the final layer adhesion (Figure 3). Figure 4 shows two examples of layers formed by using the dispersion mixtures with the inferior composition. The two layers in Figure 4 were visually non-compact and under the analytical limits of the used techniques. Other samples contained hematite, which was not a preferred phase (see Supplementary Materials).

**Figure 5:**
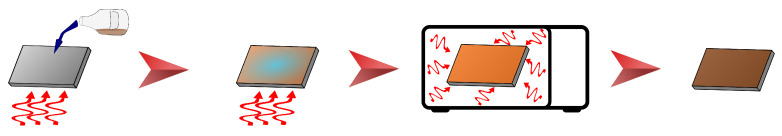
Schematic representation of covering layer formation, including drying and sintering.

A total of 30 mL of the prepared dispersion was added drop-wise on the top of the CL20ES 3D-printed cylinders preheated to 80 °C. The dispersion aided the material in spreading evenly across the surface to form the continuous film, while the surface’s roughness, created during the 3D printing and sandblasting, enhanced the adhesion. The treated cylinders were then left to dry. After the liquid evaporated, the ZnFe_2_O_4_ layer was formed. The cylinders with the formed layer were calcined in the muffle furnace at two temperatures, i.e., 350 °C or 500 °C, isothermally for 2 h. The programmed temperature rate was 3000 °C h^−1^, but the programmed temperature was reached after several minutes. The process is schematized in Figure 5.

**Figure 6:**
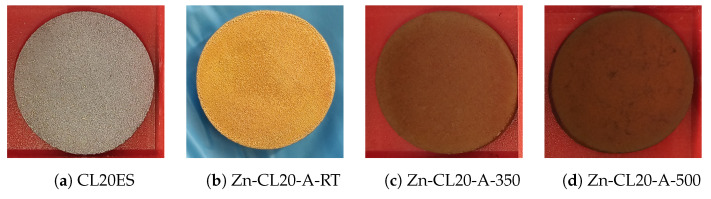
Samples of CL20ES cylinders with the zinc ferrite covering film. The diameter is 25 mm and the height is 5 mm.

**Figure 7:**
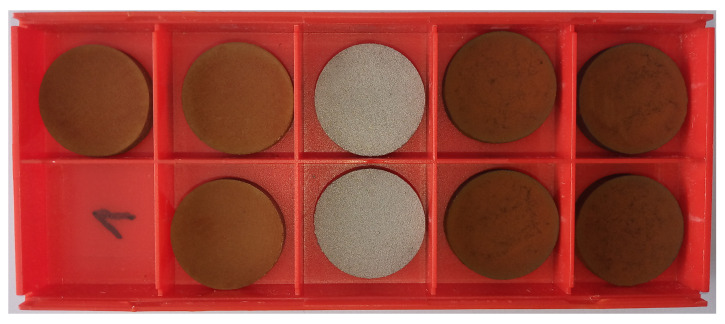
Replication of the layer preparation process: two left columns—Zn-CL20-A-350, middle—untreated, two right columns—Zn-CL20-A-500.

The cylinder’s high-resolution images at different stages of the preparation are shown in Figure 6. Images in Figure 6b–d show complete covering of the 3D-printed rough surface. The whole process was replicated to prepare multiple samples of the same kind, as shown in Figure 7.

### 2.5. X-ray Powder Diffraction

X-ray powder diffraction (XRD) was used for the determination of the crystal structure and phase composition. The employed Bruker D8 ADVANCE powder diffractometer (Bruker AXS GmbH, Karlsruhe, Germany) operates in the Bragg–Brentano para-focal geometry and is equipped with the Co K_α_ radiation source and the LYNXEYE position sensitive detector. A voltage of 35 kV and current of 40 mA were set for the X-ray tube. In addition, the 0.6 mm divergence slit and 2.5° axial Soller slits for the primary beam path and the Fe K_β_ filter and 2.5° axial Soller slits for the secondary beam path were applied during the measurements. The XRD patterns were measured in the 2θ range between 10° and 100°, with a step of 0.02°. High-temperature measurements were collected in the Anton Paar XRK 900 in situ reactor chamber, which allows different temperature programs and variable atmosphere.

Grazing incidence X-ray powder diffraction (GIXRD), the surface-sensitive method, was used as a supplement to XRD. The diffraction was measured at an incidence angle of *α* = 0.5° with a Göbel mirror in the primary path of the beam. In the secondary path, the axial slits were exchanged for the equatorial Soller slits.

XRD patterns were compared with Open Crystallography Database to determine the observed phases [37]. The measured XRD patterns were analyzed by Rietveld analysis in the MAUD program [38].

### 2.6. ^57^Fe Mössbauer Spectroscopy

To distinguish structural positions and chemical states of ^57^Fe within the crystal structure of the samples, two modes of Mössbauer spectroscopy were used. Firstly, the transmission Mössbauer spectra (TMS) were recorded with the OLTWINS dual-channel Mössbauer spectrometer [39]. Secondly, conversion electron Mössbauer spectroscopy (CEMS) was used to collect spectra specifically from the surface layer (depth ≈ 300 nm). The customs-made CEMS spectrometer works in a reflective geometry and uses a ΦЭy-85 photomultiplier as its detector. It is built into a Pfeiffer Vacuum Chamber to prolong the mean free path of the conversion electrons. The electric field is generated using an Ortec 556H High-Voltage Power Supply (ORTEC, Busan, Republic of Korea). Both TMS and CEMS modes were operated in constant acceleration mode and made use of a ^57^Co radioactive source in Rh matrix with initial activity of 50 mCi (±10%) [40]. The samples were measured at room temperature. The spectrometer’s velocity axes were calibrated using a α-Fe calibration foil. MossWinn 4.0 software was used for the spectra evaluation [41]. We used three spectra evaluation models. Zinc ferrite and austenite steel are non-magnetic with an asymmetric cubic structure, so we used a doublet model for each phase. Zinc ferrite is asymmetrical due to the nanosized samples and the austenite steel has a high amount of alloying elements deforming the elementary cell. The model was refined to contain a singlet component for austenite and a doublet for zinc ferrite in the case where zinc ferrite and austenite were fitted simultaneously. The singlet was chosen due to the small value of the quadrupole splitting energy and the overlapping of the the singlet and doublet components.

### 2.7. Scanning Electron Microscopy

VEGA3 LMU (TESCAN, Brno, Czech Republic) was used to collect the EDS spectra, equipped with Si(Li) XFlash 410 EDS detector (Bruker, Ettlingen, Germany). The primary energy of electrons was set to 30 kV. The EDS detector take-off angle was 35°. The Vega3 control software (v4.2.27.0) was used for the imaging and the elemental analysis was controlled by QUANTAX Esprit 1.9. Elemental mapping is the identification of elements within SEM images.

### 2.8. Ultrasonic Hardness Tester

Hardness tests were performed with the ultrasonic portable hardness tester MET-U1A (Centre “MET”, Moscow, Russia). The tester is equipped with a probe that operates according to the ultrasonic contact impedance method (ASTM A 1038 [42], DIN 50159) [43]. The hardness tester is precalibrated to hardness according to Brunell test type C (HRC). The hardness was measured on each side of the cylinder (treated, untreated) several times and the mean value was used to calculate the improvement in hardness as a percentage.

### 2.9. Digital Optical Microscope

The Keyence VHX-5000 optical microscope (Keyence, Osaka, Japan) was employed to provide high-resolution wide-field images of treated and untreated surfaces of the CL20ES cylinder surface with a high depth of field.

## 3. Results

### 3.1. The High-Temperature Annealing of CL20ES, ZnFe_2_O_4_ Powders, and Their Mixture

**Figure 8:**
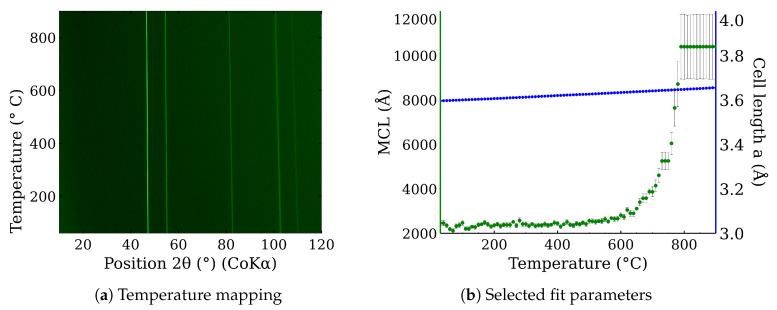
In situ high-temperature X-ray powder diffraction of CL20ES heated in dry air: (**a**) temperature mapping and (**b**) fitted parameters of the XRD patterns.

**Figure 9:**
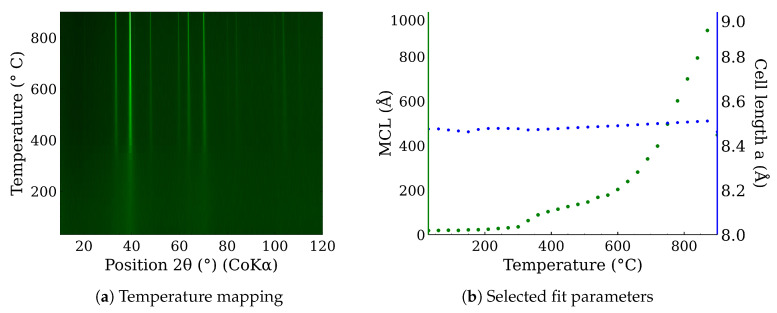
In situ high-temperature X-ray powder diffraction of the zinc ferrite heated in dry air: (**a**) temperature mapping and (**b**) fitted parameters of the XRD patterns.

In situ high-temperature X-ray powder diffraction was used to examine CL20ES phase changes in the range of temperatures from 30 °C to 900 °C in the presence of the dry air. The resultant in situ XRD map of CL20ES (Figure 8a) did not reveal any significant structural changes except for the shift in the diffraction lines, connected with the thermal expansion of the basic cell (Figure 8b). CL20ES powder is already highly crystalline and further heating changes the mean crystallite length (MCL) to the saturation. Similarly, the high-temperature in situ XRD investigation was performed for the ZnFe_2_O_4_ powder to monitor the structural changes between 30 °C and 900 °C; see Figure 9a. The results showed the gradual narrowing of the diffraction lines, which indicates the increasing crystallinity with the rising temperature. The most significant change in the relative line width was found between 300 °C and 400 °C (Figure 9b). The simple crystal growth was observed above 600 °C, where the MCL steadily increased. No other diffraction lines implying the presence of other phases were found. These measurements indicate that in the dry air, these two types of powder materials on their own are thermodynamically stable up to the high temperatures of at least 900 °C. Following these results, we prepared a series of samples for ex situ characterization, where the samples of ZnFe_2_O_4_ and CL20ES mixture were calcined at different temperatures. The calcination details can be found in the experimental section. The analyses were performed at room temperature after the thermal treatment.

**Figure 10:**
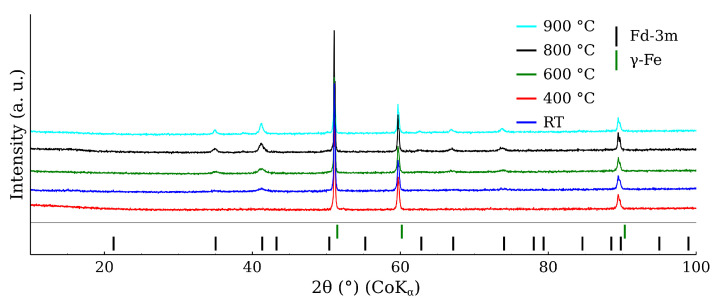
X-ray powder diffraction patterns of CL20ES steel and zinc ferrite powder mixture after the high-temperature exposure.

Figure 10 shows the XRD patterns belonging to the mixture calcined at the different temperatures. The most intensive diffraction lines were related to CL20ES, which is isostructural with γ-Fe. These three lines at 51°, 60°, and 90° exhibited significantly higher intensities than those of ZnFe_2_O_4_, owing to the higher amount of austenite (5:1 ratio) and its better crystallinity. The gradual narrowing of the ZnFe_2_O_4_ diffraction lines with the rising temperature could be observed, indicating the increasing crystallinity of the ferrite. Nonetheless, apart from these minor changes, no additional diffraction lines of any (corrosive) phase, e.g., hematite, were observed. So we can presume that there was no undesired mutual reaction between the CL20ES and ZnFe_2_O_4_ nanoparticles. The transmission Mössbauer spectroscopy was employed to exclude the formation of any amorphous phases and to monitor any changes in a close proximity of the Fe atoms.

**Figure 11:**
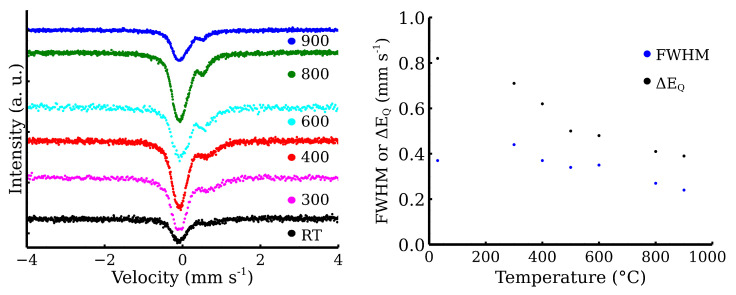
Transmission Mössbauer spectra of the steel and the zinc ferrite powder mixture after the high-temperature exposure.

The fitting model for the room-temperature Mössbauer spectra included a singlet for the austenitic stainless steel and a doublet for the zinc ferrite (Figure 11a) [11,31]. The presented transmission Mössbauer spectra exhibited significant changes in the doublet’s quadrupole splitting and full-width at half maximum (FWHM) (Table 2). The quadrupole splitting energy reflects the symmetry of Fe ion surroundings. The gradual decrease in quadrupole splitting and FWHM could be correlated with the increasing crystallinity, i.e., the increasing symmetry of Fe nuclei environment. The evolution of both parameters with the rising temperature is shown in Figure 11b). Similarly to XRD, Mössbauer spectroscopy did not show any evidence of the additional corrosive phases formation. Pre-test experiments demonstrate that a simple powder mixture is able to resist the oxidation in synthetic air at high temperatures. Therefore, zinc ferrite is a suitable material for covering layer formation on the CL20ES steel.

**Figure 12:**
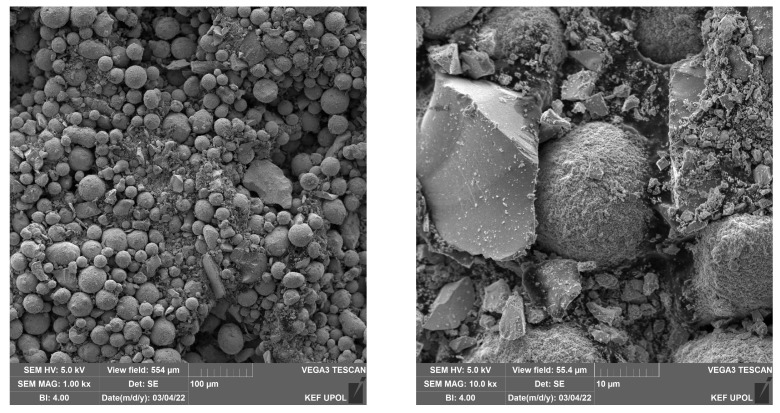
SEM images of the powder mixture heated at 900 °C.

**Figure 13:**
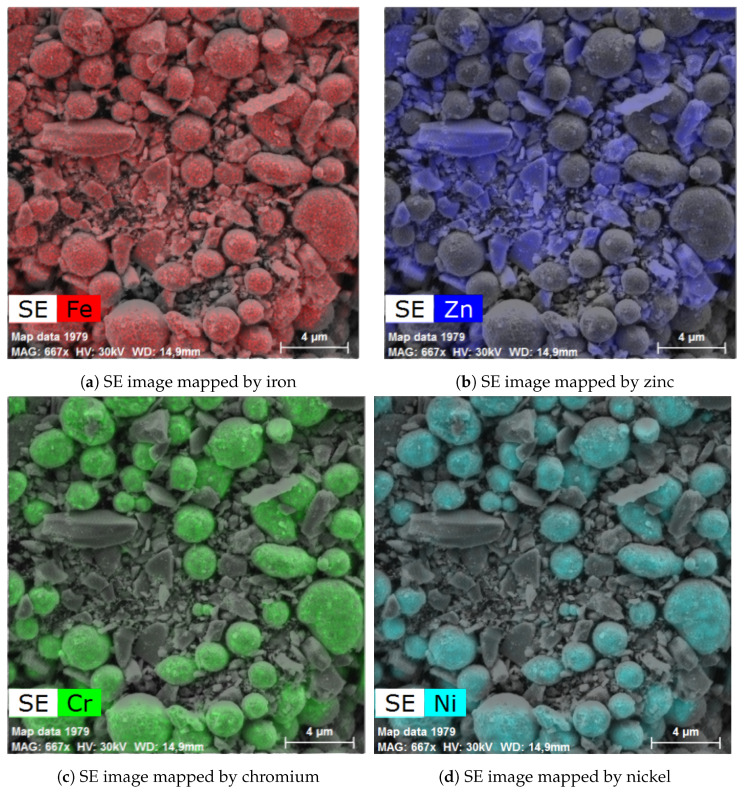
Elemental mapping and secondary electron image of the powder mixture heated at 900 °C.

The powder mixture was also analyzed by scanning electron microscope equipped with EDS and able to map elements in the acquired SEM images. The images did not show any relevant changes between the individual high-temperature treatments; see Figure 12. The additional images are provided in Supplementary Information. The provided images show two different magnifications to display both the larger area and the steel particle in detail. Figure 12 show the alloy steel CL20ES spheroidal particles, which are in the micrometer range. The zinc ferrite aggregates are surrounding those steel particles. The presented elemental maps are image fusions of the secondary electron image and the spacial distributions of the selected elements. As both CL20ES and zinc ferrite contain iron (Figure 13a), they could be distinguished by the secondary elements. The alloy steel has high amounts of Cr and Ni, while the zinc ferrite contained zinc. The chromium and nickel spacial distributions are the same and both elements were localized to the spheroidal steel particles, as seen in Figure 13c,d. Zinc was present at the complementary positions to chromium (Figure 13b).

### 3.2. ZnFe_2_O_4_ Layers on CL2OES 3D-Printed Parts

**Figure 14:**
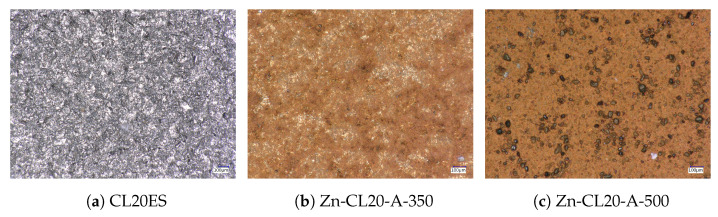
Microscopic images of the prepared CL20ES samples covered by the zinc ferrite layer.

**Figure 15:**
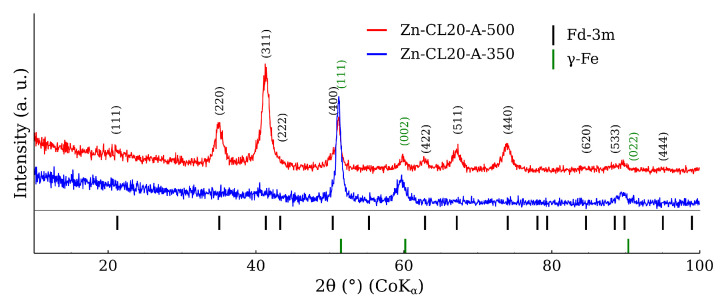
X-ray powder diffration in the graizing incidence geometry (incidence angle of 0.5°) of CL20ES plates covered by the zinc ferrite.

**Figure 16:**
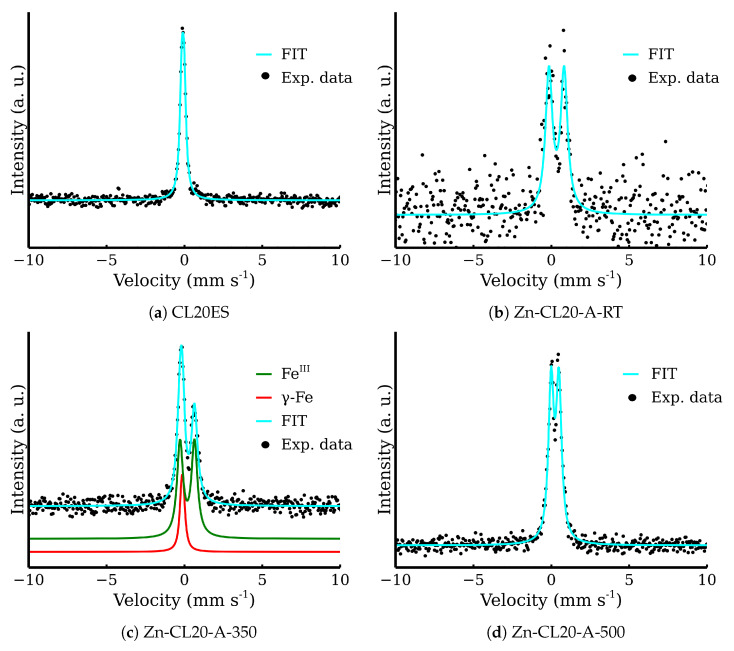
Conversion electron Mössbauer spectra of the covered steel.

The optical microscopy images at the higher magnification are shown in Figure 14. The images present the raw CL20ES cylinder and the covered samples. The layer calcined at 350 °C (Zn-CL-A-350) has grey areas belonging to CL20ES and brick-brown areas of zinc ferrite (Figure 14b). The sample calcinated at 500 °C (Zn-CL-A-500) has black spots, which are calcinated aggregates of zinc ferrite (Figure 14c). The presence and chemical integrity of the thermally treated ZnFe_2_O_4_ covering layers were verified by the surface selective methods. The preparation and thermal treatment of the ZnFe_2_O_4_ layers deposited on 3D-printed cylinders is described in the experimental section. Firstly, the X-ray powder diffraction in grazing incidence mode (GIXRD) was used to study the structural features of the surface layer. The respective diffraction patterns are shown in Figure 15 and exhibit lines of both CL20ES and ZnFe_2_O_4_. The presence of CL20ES is demonstrated by three intensive diffraction lines of austenite and the presence of zinc ferrite is ascribed to the spinel structure diffraction lines with space group Fd-3m. The presence of the spinel in the sample Zn-CL-A-350 is not clearly visible in Figure 15, where relative intensities of diffraction lines could have been influenced by the amount of the deposited ferrite and the low crystallinity. Secondly, the conversion electron Mössbauer spectroscopy (Figure 16) showed two components, a broadened doublet of zinc ferrite and a very narrow doublet ascribed to austenitic steel, which indicated (combined with XRD) the successful fabrication of the ZnFe_2_O_4_ coating. The relative intensities and signal penetration of these two methods can be used for ZnFe_2_O_4_ layer thickness estimation. The penetration depth of the electron is roughly 300 nm [40]. Based on the relative intensities, we estimate that the layer thickness of the sample calcined at 500 °C is higher than 300 nm, while the layer thickness of the other sample is lower, between roughly 100 and 200 nm. Samples Zn-CL-A-RT and Zn-CL-A-500 were fitted with only one doublet because the layer thickness was above the penetration depth (Table 3). It is evident that although both XRD and CEMS provide similar information, CEMS is more surface-sensitive than the grazing incidence XRD. The lower thickness of Zn-CL-A-350 could be caused by a lower cohesion of the zinc ferrite after the calcination, because a higher temperature may lead to a higher cohesion, sintering, and diffusion of nanoparticles.

Lastly, the ultrasonic hardness measurements showed the enhancing effect of the protective ZnFe_2_O_4_ layers, exhibiting an improvement in the surface hardness by 12% and 24%, referred to as HRC (Table 4).

## 4. Conclusions

In this paper, the process of zinc ferrite nanoparticle thin layer deposition via adhesion on austenitic stainless steel was developed. The high-temperature annealing of CL20ES powder, zinc ferrite particles, and their mixture (5:1) in dry air showed a high temperature stability of all the studied materials. Importantly, it was shown that ZnFe_2_O_4_ addition to CL20ES did not promote the formation of the corrosive phases. These experiments served as an experimental pre-test and showed that the powder mixture is able to resist oxidation in dry air at high temperature. Next, layer preparation by drop-wise technique with additional annealing and supported by a mixture of organic solvents produced a solid thin film on the 3D-printed stainless steel cylinders. The optimal quantity of all components of the dispersion mixture was found during the deposition experiments. XRD and Mössbauer spectroscopy were used in their surface-sensitive modes, i.e., grazing incidence and conversion electron detection, respectively. They proved the desired film structure with no corrosion products detected (e.g., hematite). The structure of stainless steel remained unchanged and the thin spinel zinc ferrite layer was prepared. The additional ultrasonic hardness measurement of the top-coated steel cylinders showed the improvement of the treated surface’s hardness by 24%.

## Figures and Tables

**Table 1:** Chemical composition of CL20ES in percent. The remaining percentages (up to 100%) belong to iron.

Element	Cr	Ni	Mo	Mn	Si	P	C, S
Min	16.5	10.0	2.0	0.0	0.0	0.000	0.000
Max	18.5	13.0	2.5	2.0	1.0	0.045	0.030

**Table 2:** Mössbauer spectra components in transmission geometry (TMS).

Sample	δ	$\Delta E_{Q}$	FWHM	RA	Component
	±0.02	±0.02	±0.02	±3	
	(mm s^−1^)	(mm s^−1^)	(mm s^−1^)	(%)	
Zn-CL20-RT	0.34	0.82	0.45	100	Fe^3+^
Zn-CL20-300	0.34 *	0.71	0.44	29	Fe^3+^
	−0.10	—	0.44	71	γ-Fe
Zn-CL20-400	0.34 *	0.61	0.33	26	Fe^3+^
	−0.11	—	0.41	74	γ-Fe
Zn-CL20-500	0.34 *	0.50	0.34	34	Fe^3+^
	−0.10	—	0.39	66	γ-Fe
Zn-CL20-600	0.34 *	0.48	0.35	41	Fe^3+^
	−0.11	—	0.41	59	γ-Fe
Zn-CL20-800	0.34 *	0.41	0.27	24	Fe^3+^
	−0.10	—	0.40	76	γ-Fe
Zn-CL20-900	0.34 *	0.39	0.24	22	Fe^3+^
	−0.09	—	0.40	78	γ-Fe

δ is isomer shift, Δ*E*_Q_ is quadrupole splitting energy, FWHM is full-width at half maximum, RA is relative area, and “*” stands for fixed parameter.

**Table 3:** Conversion electron reflection geometry (CEMS) spectral components.

Sample	δ	$\Delta E_{Q}$	FWHM	RA	Component
	±0.02	±0.02	±0.02	±3	
	(mm s^−1^)	(mm s^−1^)	(mm s^−1^)	(%)	
CL20ES	−0.10	0.15	0.29	100	γ-Fe
Zn-CL20-A-RT	0.34	0.97	0.53	100	Fe^3+^
Zn-CL20-A-350	−0.10	0.09	0.30	18	γ-Fe
	0.18	0.93	0.49	82	Fe^3+^
Zn-CL20-A-500	0.24	0.50	0.41	100	Fe^3+^

**Table 4:** Ultrasonic hardness test of austenitic alloy steel covering layer.

Sample	Hardness	Improvement
	±2	
	**(HRC)**	**(%)**
CL20ES	42	—
Zn-CL20-A-350	47	12
Zn-CL20-A-500	52	24

## Data Availability

Data are contained within the article and Supplementary Materials.

## Supplementary Materials

The following supporting information can be downloaded at: https://www.mdpi.com/article/10.3390/ma17040857/s1.

## References

[1] Hasegawa S., Kim S.-Y., Ebina T., Tokuda H., Ito T., Nagano N., Hitomi K., Ishii K. (2016). Effect of Nitrate on Corrosion of Austenitic Stainless Steel in Boiling Nitric Acid Solution Containing Chromium Ions. J. Nucl. Sci. Technol..

[2] Samusawa I., Shiotani K. (2015). Influence and Role of Ethanol Minor Constituents of Fuel Grade Ethanol on Corrosion Behavior of Carbon Steel. Corros. Sci..

[3] Bystrov S.G., Reshetnikov S.M., Kolotov A.A., Drozdov A.Y., Bayankin V.Y. (2021). Effect of Oxygen Ion Implantation on Physicochemical Structure and Corrosion-Electrochemical Behavior of High-Chromium Steel. Inorg. Mater. Appl. Res..

[4] Santambrogio M., Perrucci G., Trueba M., Trasatti S.P., Casaletto M.P. (2016). Effect of Major Degradation Products of Ethylene Glycol Aqueous Solutions on Steel Corrosion. Electrochim. Acta.

[5] Shi Y., Yang B., Liaw P. (2017). Corrosion-Resistant High-Entropy Alloys: A Review. J. Met..

[6] Wei L., Pang X., Gao K. (2016). Corrosion of Low Alloy Steel and Stainless Steel in Supercritical CO_2_/H_2_O/H_2_S Systems. Corros. Sci..

[7] Zhang W., Xu J. (2022). Advanced Lightweight Materials for Automobiles: A Review. Mater. Des..

[8] Liu Y., Li H., Huang S., An H., Santagata R., Ulgiati S. (2020). Environmental and Economic-Related Impact Assessment of Iron and Steel Production. A Call for Shared Responsibility in Global Trade. J. Clean. Prod..

[9] Dennis J.K., Such T.E. (1993). Control and Purification of Nickel Electroplating Solutions. Nickel and Chromium Plating.

[10] Linderhof F., Mashlan M., Doláková H., Ingr T., Ivanova T. (2021). Surface Micromorphology and Structure of Stainless and Maraging Steel Obtained via Selective Laser Melting: A Mössbauer Spectroscopy Study. J. Met..

[11] Ivanova T., Kořenek M., Mashlan M., Svačinová V. (2023). Mössbauer Study of Thermal Behavior of CL20ES and CL50WS Steel Powders Used in Selective Laser Melting. Chem. Pap..

[12] Zhong J.-Y., Sun J.-Y., Liu D.-B., Li X.-G., Liu T.-Q. (2010). Effects of Chromium on the Corrosion and Electrochemical Behaviors of Ultra High Strength Steels. Int. J. Miner. Metall. Mater..

[13] Kashima K., Sugae K., Kamimura T., Miyuki H., Kudo T. (2013). Effect of Chromium Contents on Atmospheric Corrosion of Steel in Chloride Environment. J. Jpn. Inst. Met..

[14] Wint N., de Vooys A.C.A., McMurray H.N. (2016). The Corrosion of Chromium Based Coatings for Packaging Steel. Electrochim. Acta.

[15] Kamimura T., Stratmann M. (2001). The Influence of Chromium on the Atmospheric Corrosion of Steel. Corros. Sci..

[16] Rezaee N., Attar M.M., Ramezanzadeh B. (2013). Studying Corrosion Performance, Microstructure and Adhesion Properties of a Room Temperature Zinc Phosphate Conversion Coating Containing Mn^2+^ on Mild Steel. Surf. Coat. Technol..

[17] Maurice V., Marcus P. (2018). Current Developments of Nanoscale Insight into Corrosion Protection by Passive Oxide Films. Curr. Opin. Solid State Mater. Sci..

[18] Jiang C., Gao Z., Pan H., Cheng X. (2020). The Initiation and Formation of a Double-Layer Phosphate Conversion Coating on Steel. Electrochem. Commun..

[19] Ujiro T., Yoshioka K., Staehle R.W. (1994). Differences in Corrosion Behavior of Ferritic and Austenitic Stainless Steels. Corrosion.

[20] Klapper H.S., Burkert A., Burkert A., Lehmann J., Villalba A.L. (2011). Influence of Surface Treatments on the Pitting Corrosion of Type 304 Stainless Steel by Electrochemical Noise Measurements. Corrosion.

[21] Hoshino K., Furuya S., Buchheit R.G. (2019). Effect of Solution Ph on Layered Double Hydroxide Formation on Electrogalvanized Steel Sheets. J. Mater. Eng. Perform..

[22] Ramezanzadeh B., Vakili H., Amini R. (2015). The Effects of Addition of Poly(Vinyl) Alcohol (PVA) as a Green Corrosion Inhibitor to the Phosphate Conversion Coating on the Anticorrosion and Adhesion Properties of the Epoxy Coating on the Steel Substrate. Appl. Surf. Sci..

[23] Buchheit R.G., Guan H., Mahajanam S., Wong F. (2003). Active Corrosion Protection and Corrosion Sensing in Chromate-Free Organic Coatings. Prog. Org. Coat..

[24] Winn D., Dalton W. (2008). Chromium-Free Corrosion Solutions. Met. Finish..

[25] Jing C., Dong B., Raza A., Zhang T., Zhang Y. (2021). Corrosion Inhibition of Layered Double Hydroxides for Metal-Based Systems. Nano Mater. Sci..

[26] Li X., Sun W., Zheng Y., Long C., Wang Q. (2023). New Strategy for the Design of Anti-Corrosion Coatings in Bipolar Plates Based on Hybrid Organic–Inorganic Layers. Molecules.

[27] Nguyen T.D., Tran B.A., Vu K.O., Nguyen A.S., Trinh A.T., Pham G.V., To T.X., Phan M.V., Phan T.T. (2018). Corrosion Protection of Carbon Steel Using Hydrotalcite/Graphene Oxide Nanohybrid. J. Coat. Technol. Res..

[28] Boinovich L.B., Gnedenkov S.V., Alpysbaeva D.A., Egorkin V.S., Emelyanenko A.M., Sinebryukhov S.L., Zaretskaya A.K. (2012). Corrosion Resistance of Composite Coatings on Low-Carbon Steel Containing Hydrophobic and Superhydrophobic Layers in Combination with Oxide Sublayers. Corros. Sci..

[29] Imran Din M., Rafique F., Hussain M.S., Arslan Mehmood H., Waseem S. (2019). Recent Developments in the Synthesis and Stability of Metal Ferrite Nanoparticles. Sci. Prog..

[30] Hasirci C., Karaagac O., Köçkar H. (2019). Superparamagnetic Zinc Ferrite: A Correlation between High Magnetizations and Nanoparticle Sizes as a Function of Reaction Time via Hydrothermal Process. J. Magn. Magn. Mater..

[31] Ochmann M., Vrba V., Kopp J., Ingr T., Malina O., Machala L. (2022). Microwave-Enhanced Crystalline Properties of Zinc Ferrite Nanoparticles. J. Nanomater..

[32] Ochmann M., Linderhof F.M., Machala L. Spinel Ferrites Nanoparticles for Alloy Steel Protective Layers. Proceedings of the 12th International Conference on Nanomaterials-Research & Application.

[33] Pitrmuc Z., Šimota J., Beránek L., Mikeš P., Andronov V., Sommer J., Holešovský F. (2022). Mechanical and Microstructural Anisotropy of Laser Powder Bed Fusion 316L Stainless Steel. Materials.

[34] Gao Z., Zhang D., Li X., Jiang S., Zhang Q. (2018). Current Status, Opportunities and Challenges in Chemical Conversion Coatings for Zinc. Colloids Surf..

[35] Saei E., Ramezanzadeh B., Amini R., Kalajahi M.S. (2017). Effects of Combined Organic and Inorganic Corrosion Inhibitors on the Nanostructure Cerium Based Conversion Coating Performance on az31 Magnesium Alloy: Morphological and Corrosion Studies. Corros. Sci..

[36] Holzner T., Luckeneder G., Strauss B., Valtiner M. (2022). Environmentally Friendly Layered Double Hydroxide Conversion Layers: Formation Kinetics on Zn-Al-Mg-Coated Steel. ACS Appl. Mater. Interfaces.

[37] Gražulis S., Chateigner D., Downs R.T., Yokochi A.F., Quirós M., Lutterotti L., Manakova E., Butkus J., Moeck P., Le Bail A. (2009). Crystallography Open Database—An Open-Access Collection of Crystal Structures. J. Appl. Crystallogr..

[38] Lutterotti L. (2010). Total Pattern Fitting for the Combined Size–Strain–Stress–Texture Determination in Thin Film Diffraction. Nucl. Instrum. Methods Phys. Res. B.

[39] Stejskal A., Procházka V., Dudka M., Vrba V., Kočiščák J., Šretrová P., Novák P. (2023). A Dual Mössbauer Spectrometer for Material Research, Coincidence Experiments and Nuclear Quantum Optics. Measurement.

[40] Pechoušek J., Jančík D., Frydrych J., Navařík J., Novák P. (2012). Setup of Mössbauer Spectrometers at RCPTM. AIP Conf. Proc..

[41] Klencsár Z. (1997). Mössbauer Spectrum Analysis by Evolution Algorithm. Nucl. Instrum. Methods Phys. Res. B.

[42] Standard Test Method for Portable Hardness Testing by the Ultrasonic Contact Impedance Method. https://www.astm.org/a1038-19.html.

[43] (2022). Metallic Materials—Hardness Testing with the UCI Method—Part 1: Test Method.

