# Supplementary material for: Zinc Ferrite Nanoparticle Coatings on Austenitic Alloy Steel
Source: Materials (Basel). 2024 Feb 12;17(4):857. doi: 10.3390/ma17040857 (PMC10890098; doi:10.3390/ma17040857)
Supplement: Supplementary file 1 [file materials-17-00857-s001.zip › materials-2807244-supplementary.pdf]

1. Prepared layer with undesired hematite

The layer was prepared by a drop-wise deposition of the zinc ferrite powder dispersed in isopropyl alcohol. During the deposition the CL20ES plate was pre-heated at 80 °C for higher evaporation of alcohol. Finally, the plate was heated in the muffle furnace LE 05/11 HT 40P (LAC, Rajhrad, Czech Republic) at 400 °C for 2 hours. The formed layer had brown-brick colour associated with zinc ferrite (Fig. S1). The layer was analyzed by the conversion electron Mössbauer spectroscopy, which revealed the hematite presence in the depth less then 300 nm (Fig. S2).

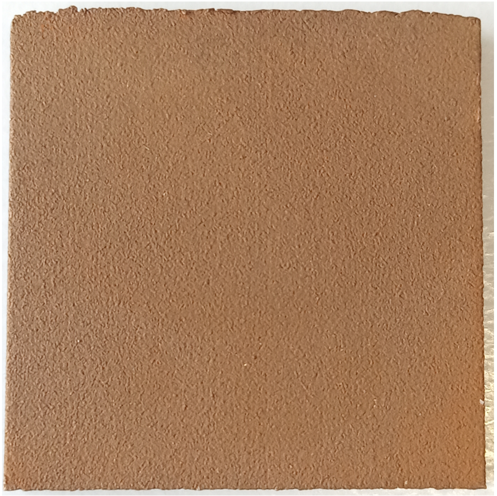

Figure S1. Layer formed from the zinc ferrite dispersed in isopropyl alcohol on the CL20ES plate.

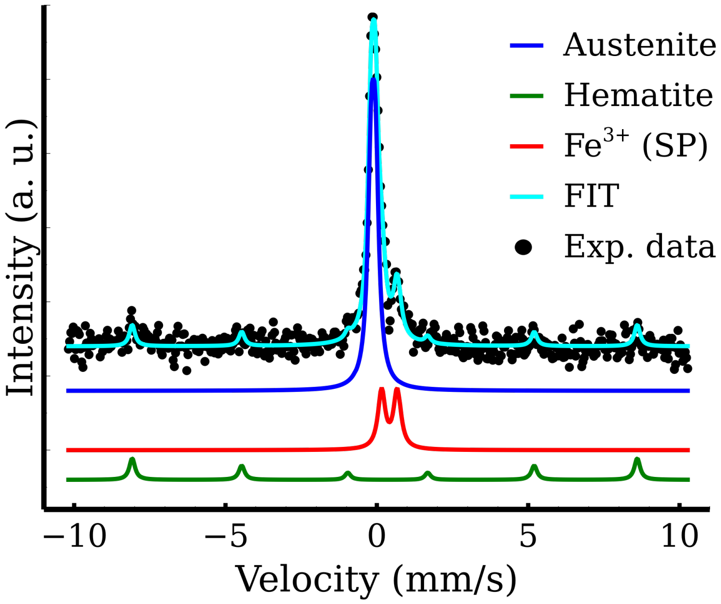

Figure S2. CEMS of layer formed from the zinc ferrite dispersed in isopropyl alcohol on the CL20ES plate.

2. Transmission ^{57}Fe Mössbauer spectra

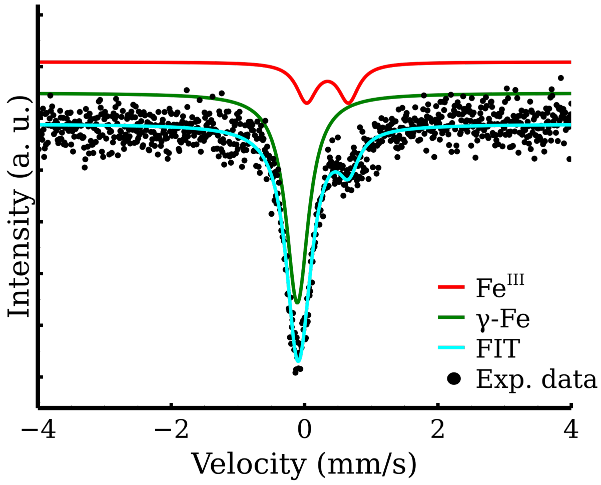

Figure S3. Transmission Mössbauer spectrum of the steel and the zinc ferrite powder mixture.

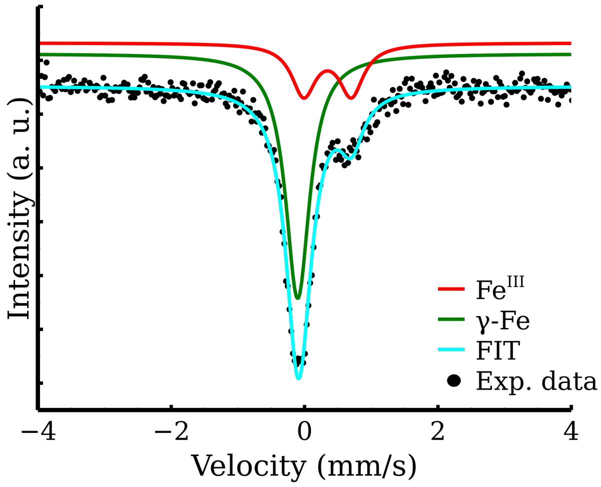

Figure S4. Transmission Mössbauer spectrum of the steel and the zinc ferrite powder mixture heated at 300 °C.

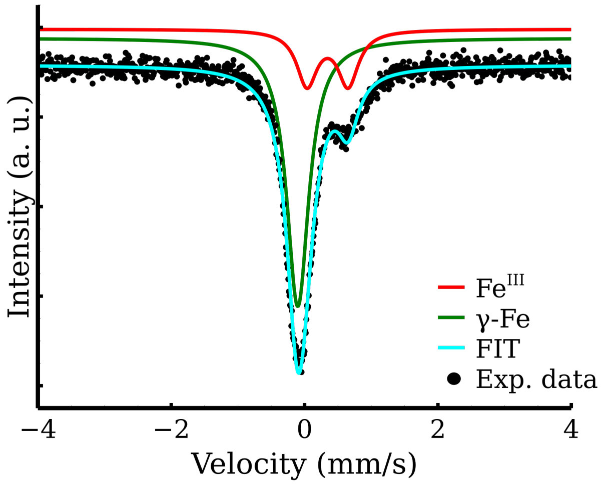

Figure S5. Transmission Mössbauer spectrum of the steel and the zinc ferrite powder mixture heated at 400 °C.

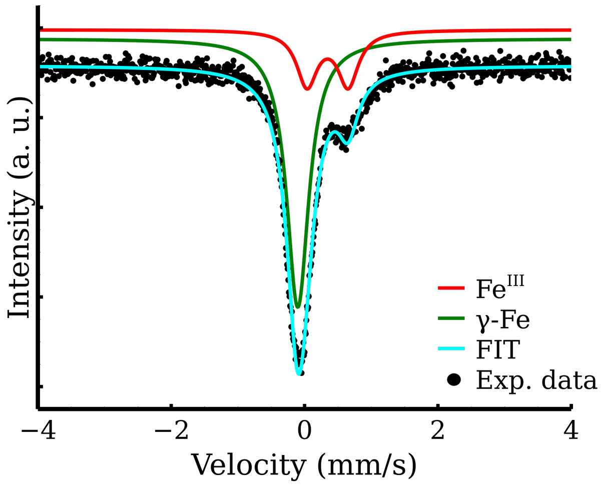

Figure S6. Transmission Mössbauer spectrum of the steel and the zinc ferrite powder mixture heated at 500 °C.

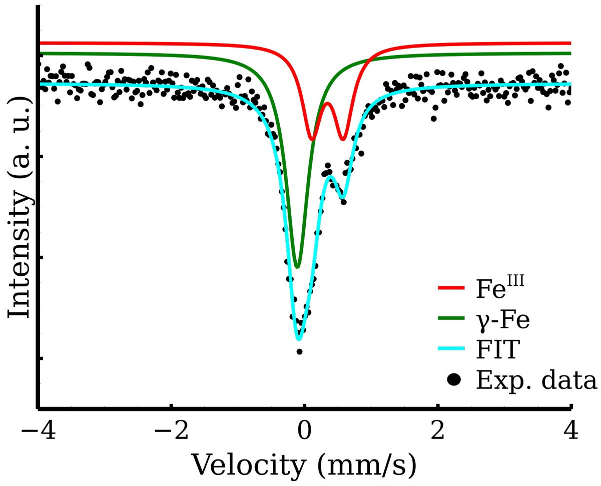

Figure S7. Transmission Mössbauer spectrum of the steel and the zinc ferrite powder mixture heated at 600 °C.

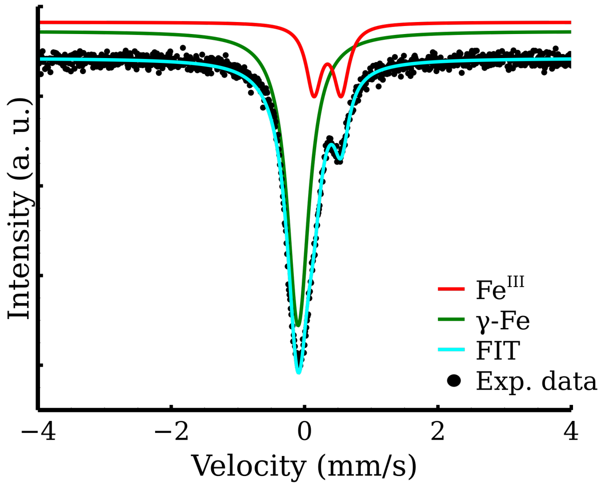

Figure S8. Transmission Mössbauer spectrum of the steel and the zinc ferrite powder mixture heated at 800 °C.

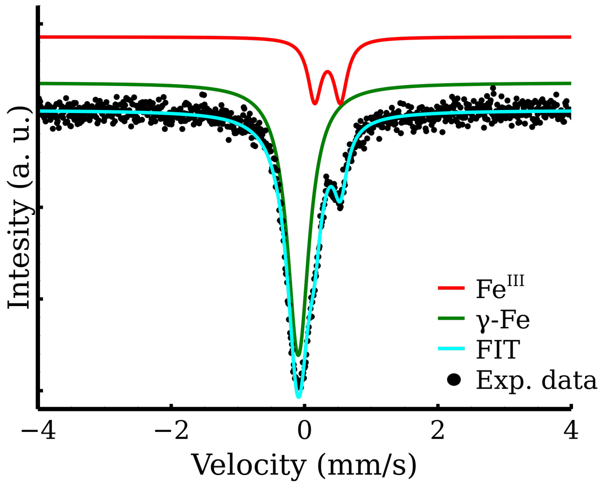

Figure S9. Transmission Mössbauer spectrum of the steel and the zinc ferrite powder mixture heated at 900 °C.

3. SEM images

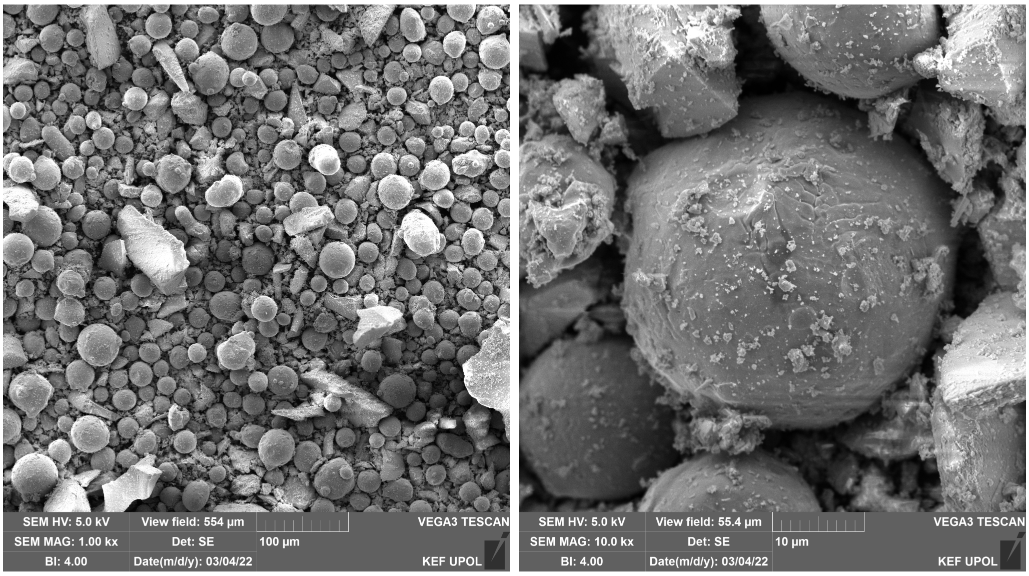

Figure S10. SEM images of the powder mixture.

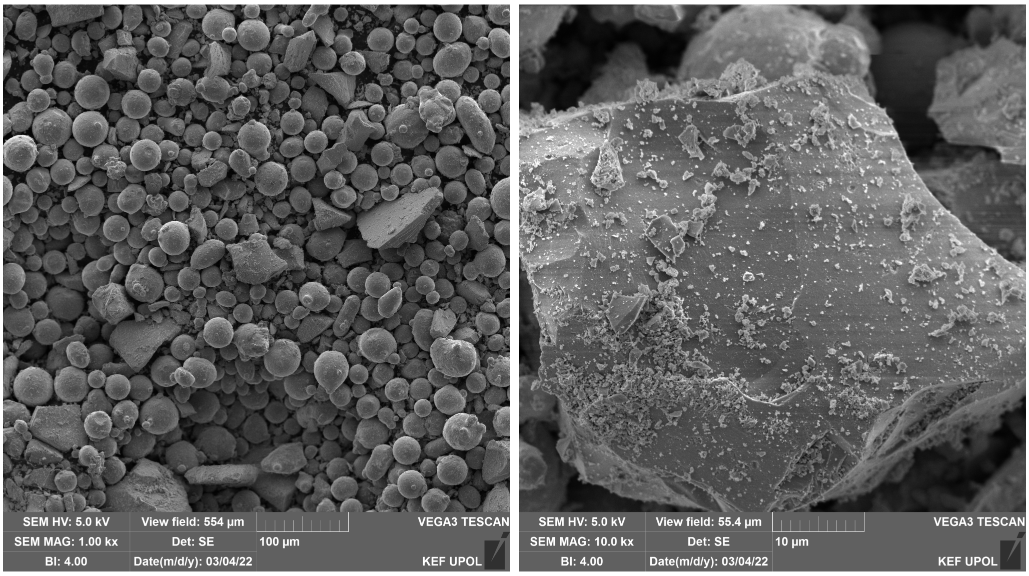

Figure S11. SEM images of the powder mixture heated at 400 °C.

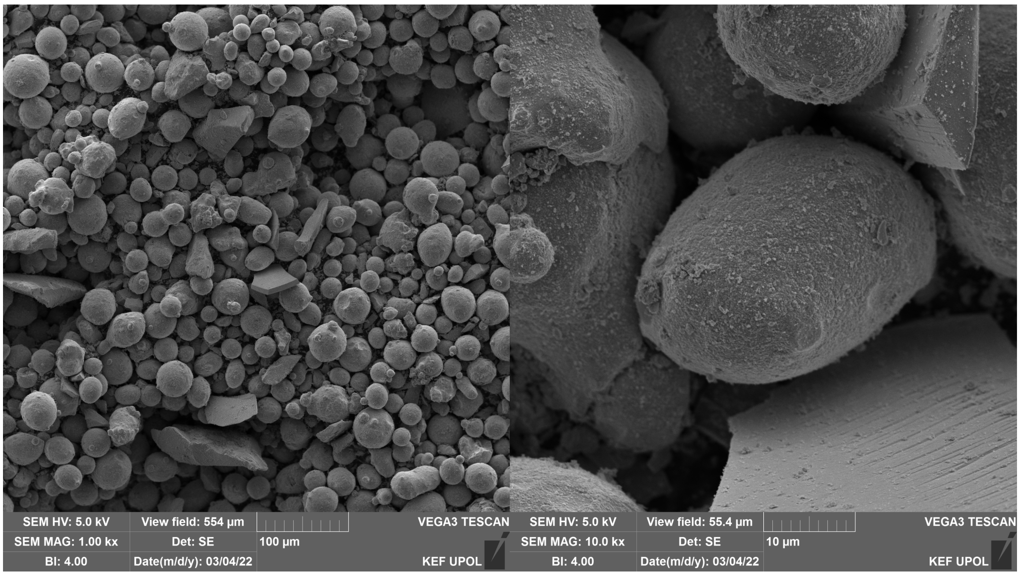

Figure S12. SEM images of the powder mixture heated at 600 °C.

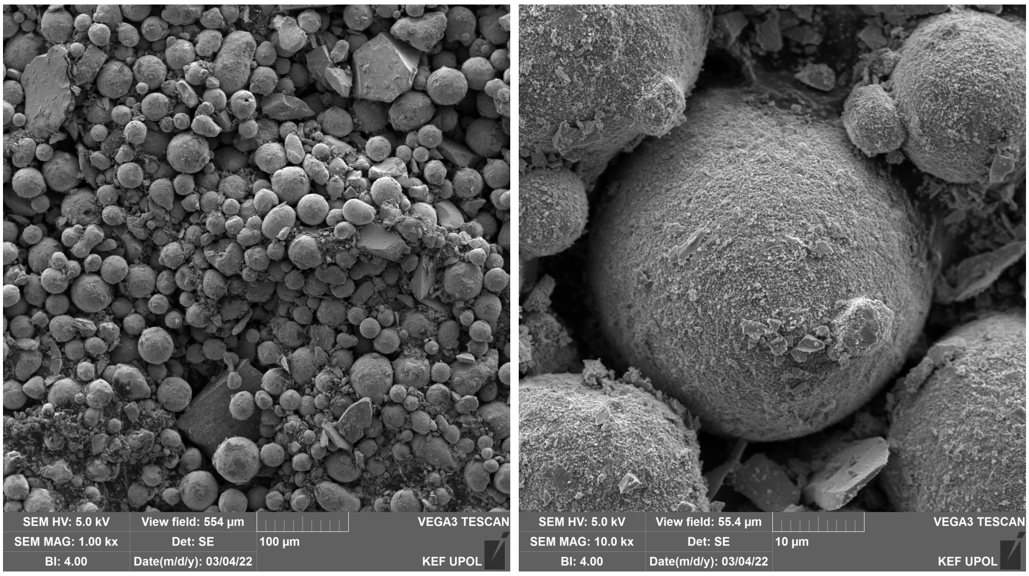

Figure S13. SEM images of the powder mixture heated at 800 °C.

4. SEM images with the elemental mapping

The steel and the zinc ferrite are containing iron so the primarily displayed iron (in red) is overlapped by the characteristic elements. Zinc and chromium were chosen to be displayed for visual separation of these phases. The chromium spacial distribution could be associated with the alloy steel while the spacial distribution of zinc could be related to the zinc ferrite.

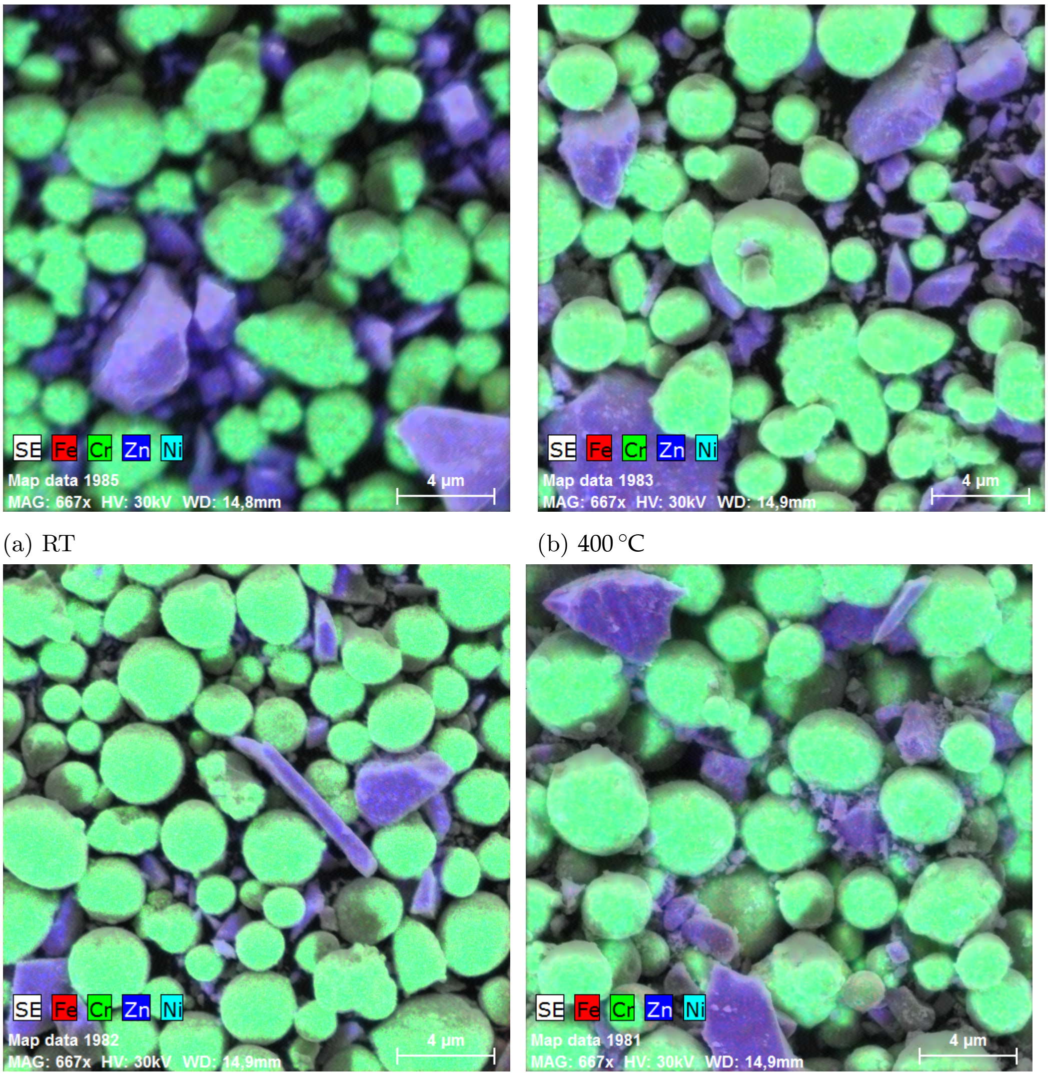

(a) RT

(b) 400 °C

(c) 600 °C

(d) 800 °C

Figure S14. SEM images with elemental mapping of powder mixture.
